# Eicosapentaenoic acid reduces the proportion of IL-17A–producing T cells in a 3D psoriatic skin model

**DOI:** 10.1016/j.jlr.2023.100428

**Published:** 2023-08-18

**Authors:** Sophie Morin, Sarah Bélanger, Sergio Cortez Ghio, Roxane Pouliot

**Affiliations:** 1 Center for Research in Experimental Organogenesis of Laval University/LOEX, Regenerative Medicine Axis, CHU of Quebec/Laval University Research Center, Qu ebec, QC, Canada; 2 Faculty of Pharmacy, Laval University, Quebec, QC, Canada; 3 Ex Machina Biostats, Research Support Department, Quebec, QC, Canada

**Keywords:** skin, psoriasis, omega-3 fatty acids, inflammation, lymphocytes, cell signaling, in vitro model

## Abstract

Psoriasis is a skin disease presenting as erythematous lesions with accentuated proliferation of epidermal keratinocytes, infiltration of leukocytes, and dysregulated lipid metabolism. T cells play essential roles in the disease. n-3 polyunsaturated fatty acids are anti-inflammatory metabolites, which exert an immunosuppressive effect on healthy T cells. However, the precise mechanistic processes of n-3 polyunsaturated fatty acids on T cells in psoriasis are still unrevealed. In this study, we aimed to evaluate the action of eicosapentaenoic acid (EPA) on T cells in a psoriatic skin model produced with T cells. A coculture of psoriatic keratinocytes and polarized T cells was prepared using culture media, which was either supplemented with 10 μM EPA or left unsupplemented. Healthy and psoriatic skin substitutes were produced according to the self-assembly method. In the coculture model, EPA reduced the proportion of IL-17A–positive cells, while increasing that of FOXP3-positive cells, suggesting an increase in the polarization of regulatory T cells. In the 3D psoriatic skin model, EPA normalized the proliferation of psoriatic keratinocytes and diminished the levels of IL-17A. The expression of the proteins of the signal transducer and activator of transcription was influenced following EPA supplementation with downregulation of the phosphorylation levels of signal transducer and activator of transcription 3 in the dermis. Finally, the NFκB signaling pathway was modified in the EPA-supplemented substitutes with an increase in Fas amounts. Ultimately, our results suggest that in this psoriatic model, EPA exerts its anti-inflammatory action by decreasing the proportion of IL-17A–producing T cells.

Psoriasis is a chronic inflammatory skin disease that presents as reddish, well-defined, erythematous lesions (1, 2). The main histological manifestations of the pathology are acanthosis, which presents as an increase in the thickness of the epidermis that is linked to an accentuated proliferation of psoriatic keratinocytes (3, 4). The disease also comprises an abnormal and incomplete differentiation of keratinocytes with parakeratosis, the retention of the nuclei in the stratum corneum of the epidermis (5). In addition, psoriasis is characterized by a significant inflammatory infiltration of leukocytes, mainly in the dermis but also in the epidermis of the skin. The main infiltrating cells are dendritic cells, monocytes, and T cells (6, 7). In fact, T cells play key roles in the pathology, since they are at the origin of the vicious loop of cell activation, inducing psoriatic lesions and promoting their persistence (8). The pathogenesis of psoriasis is driven by the dysfunction of T cell subsets, including an increase in helper T cell (Th) Th1, Th22, and Th17 function. In fact, there is an important alteration in the balance between Th17 cells and regulatory T cells (Tregs), with a predominance of Th17 cells generating a proinflammatory environment (7, 9). In the skin, the different subpopulations of Th cells are characterized by their specific secretory profile in cytokines. Th1 cells are recognized for secreting interferon-γ (IFN-γ), Th17 cells are major producers of interleukin-17A (IL-17A), a highly inflammatory cytokine, while Treg cells secrete interleukin-10 (IL-10) and act as regulators of inflammation, allowing the return to homeostasis (10). It should be pointed out that IL-17A can also be produced by gamma delta T cells, as well as by cytotoxic T cells (11). Additionally, the signal transducer and activator of transcription (STAT) protein family plays key roles in cytokine signaling and Th cell differentiation in psoriasis. The STAT proteins are latent cytoplasmic transcription factors that upregulate the transcription of different target genes involved in cell proliferation and angiogenesis (12, 13). Among the seven different isoforms that exist, STAT1 is essential for Th1 cell signaling, while STAT3 is implicated in the differentiation and polarization of Th17 cells (14, 15). Other factors can influence naive T cell activation and polarization in psoriasis, including the NFκB signaling pathway that mediates T cell signaling and activation (16).

Moreover, the proinflammatory phenotype of psoriasis is accompanied by oxidative stress and an increased lipid metabolism of phospholipids. In fact, lipid mediators derived from essential fatty acids play important roles in the pathophysiology of psoriasis as they modulate the activity of immune cells, among other things (17). n-3 and n-6 polyunsaturated fatty acids (PUFAs) are the main sources of bioactive lipid mediators in the skin (18). In membrane phospholipids of psoriatic skin, accumulation of arachidonic acid, a n-6 PUFA metabolite, leads to an increase in oxylipins produced by cyclooxygenases and lipoxygenases, which are linked to proinflammatory functions. This is accompanied by a decrease in eicosapentaenoic acid (EPA) and docosahexaenoic acid (DHA)-derived lipid mediators, which rather exert anti-inflammatory and immunomodulating actions (19, 20, 21). This modulation of lipid mediators in psoriatic skin, along with the increased production of n-6 PUFA–inflammatory derivatives, accelerates the secretion of proinflammatory cytokines in the skin (21, 22). Inversely, n-3 PUFAs modulate the immune response by limiting inflammation. Because of their competitive metabolism with n-6 PUFAs, n-3 PUFAs can inhibit the production of proinflammatory bioactive lipid mediators. They are also able to modify the membrane fluidity of immune cells, modulate the signaling cascades by binding to different G protein–coupled receptor and nuclear receptors, as well as produce proresolving mediators (23, 24). T cell activation and polarization into Th subsets can be controlled by n-3 PUFAs to avoid T cell hyperactivation. In fact, studies in mice have shown that an n-3 PUFA–enriched diet increases the quantity of Th2 polarized cells while suppressing the number of Th1 cells (25). In the study by Mizota *et al.* (26), feeding mice with n-3 PUFA supplements resulted in a significant decrease in the IFN-γ/ interleukin-4 (IL-4) ratio, indicating a decrease in Th1 cell polarization. Finally, elegant data by Monk *et al.* (27) also showed that n-3 PUFAs reduce ex vivo Th17 polarization in healthy mice. However, the precise effects of n-3 PUFAs on T cell polarization in the human psoriatic environment are still not known to this day.

In the past decade, a significant amount of research has been conducted on testing the beneficial potential of n-3 PUFAs to improve the symptoms of psoriasis. Most studies found that a diet rich in n-3 PUFAs improves the psoriasis area and severity index score, erythema, scaling, and itching of the skin, which are hallmarks of psoriasis (28, 29). Our team has previously tested the effects of a supplementation of the culture media with alpha-linolenic acid (ALA), the precursor of n-3 PUFAs, on the main psoriatic characteristics using our psoriatic skin model produced with activated T cells. Despite the fact that this model uses T cells isolated from healthy donors, interactions between these activated T cells and psoriatic keratinocytes create an amplified proinflammatory environment characteristic of the disease. The use of the same T cell population for the reconstruction of both healthy and psoriatic skin substitutes has previously demonstrated that the presence of psoriatic keratinocytes is essential in order to activate the response of inflammatory T cells (30). We then found that ALA decreased the proliferation of psoriatic keratinocytes as well as the secretion of T cell–related inflammatory cytokines. Additionally, lipid analyses showed that ALA was incorporated into the phospholipid fraction of the epidermis and mainly metabolized into EPA (31). Therefore, since the bioaction of EPA on the inflammation profile of psoriasis is still unknown, we wanted to further evaluate the influence of a supplementation of the culture media with EPA on our psoriatic model produced with polarized T cells. Consequently, the main objective of the present study was to determine the effects of EPA on the T cell phenotype specifically in the psoriatic context. The current data were also intended to determine the bioactivity of EPA on psoriatic characteristics that are managed by polarized T cells, mainly the expression of the STAT proteins, the secretion of cytokines, as well as the regulation of the NFκB signaling pathway.

## Materials and methods

### Biopsies and ethics

The present study was conducted according to the Declaration of Helsinki and approved by the Research Ethics Committee of the Centre Hospitalier Universitaire de Québec-Université Laval. The production of tissue-engineered healthy skin substitutes was performed using skin cells (fibroblasts and keratinocytes) from breast reduction skin biopsies of three Caucasian women aged 18, 38, and 46 years old. As for psoriatic skin substitutes, they were produced with skin biopsies extracted from patients with plaque psoriasis aged 36 (woman, back biopsy, 5–10% psoriasis extent, no treatment), 39 (man, psoriasis area and severity index score of 17), and 64 (woman, back biopsy, 20% psoriasis extent, no treatment) years old, respectively. All donors were given adequate information and provided written consent.

### Cell culture conditions

Fibroblasts were cultured in the DMEM with a supplementation of 10% bovine growth serum (FB Essence; Seradigm, Mississauga, ON, Canada), 100 Ul/ml penicillin G (Sigma, Oakville, ON, Canada), and 25 μg/ml gentamicin (Gemini Bio-Products, Sacramento, CA). Keratinocytes were cultured in a combination of DMEM with Ham’s F12 (3:1), supplemented with 5% FetalClone II serum (Galenova, Saint-Hyacinthe, QC, Canada), 5 μg/ml insulin (Sigma, Oakville, ON, Canada), 0.4 μg/ml hydrocortisone (Galenova, St-Hyacinthe, QC, CA), 10^-10^ M cholera toxin (Sigma, Oakville, ON, Canada), 10 ng/ml human epidermal growth factor (Ango Inc, San Ramon, CA), 100 Ul/ml penicillin G (Sigma, Oakville, ON, Canada), and 25 μg/ml gentamicin (Gemini Bio-Products, Sacramento, CA). EPA supplementation was provided by dissolving EPA (Cedarlane, Burlington, ON, Canada) in ethanol in order to generate a stock solution. The EPA solution was then added to the serum to provide a concentration of 10 μM EPA, after evaporation of the remaining ethanol (32, 33).

### T cell isolation, activation, and polarization

T cells were isolated using the EasySep™ Direct Human T Cell Isolation Kit (StemCell Technologies, Vancouver, BC, Canada) following the manufacturer’s instructions. Using this technique, T cells were separated from human anticoagulated whole blood obtained from healthy donors (blood donors were not matched with the psoriatic cell donors) (30). Blood samples were obtained from three healthy donors without health problems identified during the blood test, respectively aged 33 (man), 47 (man), and 48 (woman) years old. All blood samples were obtained in agreement with the Declaration of Helsinki and approved by the Research Ethics Committee of the Centre Hospitalier Universitaire de Québec-Université Laval. Once correctly isolated, the T cell population was seeded in 12-well plates at a concentration of 2 × 10^6^ cells/ml per well and separately polarized either toward a Th1 phenotype by supplementing the media with 20 ng/ml IL-12 and 5 μg/ml anti-IL-4 or toward a Th17 phenotype by supplementing the media with 10 μg/ml anti-IFN-γ, 5 μg/ml anti-IL-4, 20 ng/ml IL-1β, and 40 ng/ml IL-6. The polarized Th1 and Th17 cells were incubated at 37°C for 3 days. After the incubation period, the Th1 and Th17 cells harvested from the supernatants were washed with DMEM and independently activated with both 25 ng/ml PMA (Sigma, St. Louis, MO) and 1 μg/ml ionomycin (Sigma, St. Louis, MO) for 4 h at 37°C (34). After the activation period, the Th1 and Th17 cells were reseeded into 12-well plates (2 × 10^6^ cells/ml per well) for an additional proliferation period of 4 days at 37°C under 8% CO_2_ with the addition of IL-2 (30 U/ml R&D Systems, Burlington, ON, Canada) for Th1 cells and with the addition of IL-2 (10 U/ml) and IL-23 (20 ng/ml, BioLegend, San Diego, CA) for Th17 cells. Then the T cells were seeded onto dermal sheets at a total concentration of 0.5 × 10^6^ cells per psoriatic skin substitute (0.25 × 10^6^ Th1 polarized cells and 0.25 × 10^6^ Th17 polarized cells) (34).

### 2D coculture of psoriatic keratinocytes and T cells

At day 1, psoriatic keratinocytes at passage 3 were seeded into 6-well plates at a concentration of 4 × 10^5^ cells/well onto a layer of irradiated human fibroblasts (previously seeded at a density of 8 × 10^3^ cells per cm^2^ and cultured over a period of 1 week in order to reach the confluence). Keratinocytes were cultured in medium supplemented or not with 10 μM EPA. Two days later, isolated T cells were seeded onto the keratinocytes (2 × 10^6^ cells/well) and separately polarized either toward a Th1 or a Th17 profile as explained in T cell isolation, activation, and polarization. After 3 days, the Th1 and Th17 cells harvested from the supernatants were activated as previously reported and reseeded onto keratinocytes at an equal ratio (50/50) of Th1 and Th17 cells and cultured for 6 more days in medium supplemented or not with 10 μM EPA. The 2D coculture was maintained under 8% CO_2_ and at 37°C, and the culture media were changed every two days. To do so, the culture media were collected and centrifuged at 700 rcf to retrieve T cells in the pellet. Then the T cells were resuspended in fresh medium supplemented with the appropriate cytokines (with or without EPA supplementation) and reseeded into the 6-well plates. At day 12, T cells harvested from the supernatants were collected and analyzed by flow cytometry. In parallel, the same experiment was redone to perform flow cytometry analyses on complete populations of T cells (those present in the supernatants and those having adhered to the keratinocytes). To do so, at the end of cell culture, the cells were detached from the 6-well plates using a PBS-EDTA (2 mM) solution (10 min on ice), followed by mechanical harvesting of the cells (see Supplemental Fig. S4).

### Production of 3D tissue–engineered skin substitutes

Healthy and psoriatic skin substitutes were produced according to the self-assembly method (30, 35). Briefly, human fibroblasts were seeded into 6-well plates at a concentration of 1.2 × 10^4^ cells/well and maintained in submerged culture condition for a total of 28 days. The cells were kept at 37°C and under 8% CO_2_ with the addition of ascorbic acid in order to form manipulable dermal sheets, through the cells’ own secretion of extracellular matrix. Half of the dermal sheets were seeded with 0.5 × 10^6^ activated and polarized T cells each (detailed in T cell isolation, activation, and polarization), while the remaining dermal sheets were seeded with 1.2 × 10^6^ keratinocytes per sheet. Both dermal assemblies were cultured separately in submerged conditions over a week in the presence of 10 U/ml recombinant human IL-2 and 20 ng/ml IL-23 for the dermal sheet with T cells. The culture media were changed every day for the dermal sheet with keratinocytes, and once in every two days for the sheet with T cells. Then the air-liquid assembly was performed by stacking the sheet of fibroblasts and keratinocytes on top of that containing fibroblasts and activated/polarized T cells. Each skin equivalent was then raised to the air-liquid interface, which allowed keratinocytes to correctly differentiate. The skin substitutes were cultured in the air-liquid assembly for a total of 21 days (see supplemental Fig. S1) (30).

### Flow cytometry analysis on 2D coculture

After the 2D coculture of psoriatic keratinocytes and T cells, all T cells were harvested from the supernatants and used for flow cytometry analyses. In parallel, complete populations of T cells (those present in the supernatants and those having adhered to the keratinocytes) were also collected and used for flow cytometry analyses (see supplemental Fig. S4). An equal ratio of Th1 and Th17 cells was used for flow cytometry analyses. T cells were resuspended at a concentration of 1 × 10^6^ cells/ml in flow cytometry cell staining buffer (BioLegend, San Diego, CA). Intracellular and cell surface staining were performed using different combinations of APC-anti-CD3 (BioLegend, San Diego, CA), FITC-anti-CD69 (BioLegend, San Diego, CA), phycoerythrin (PE)-anti-IL-17A (BioLegend, San Diego, CA), PE-anti-FOXP3 (Invitrogen, Waltham, MA), and PECy7-anti-IFNγ (BioLegend, San Diego, CA). The intracellular staining of IL-17A, IFN-γ, and FOXP3 was done on T cells after stimulation for 4 h with PMA, ionomycin, and brefeldin A (blocking agent) (BioLegend, San Diego, CA). The staining was performed as follows: cells were stained with CD3 and CD69 at room temperature for 20 min in the dark. Then the cells were fixed and permeabilized for 20 min at 4°C, followed by 30 min of staining with the intracellular marker (IL-17A, IFN-γ, or FOXP3). The antibodies used are detailed in the supplementary material (see supplemental Table S1). The fluorescence measurements were done using a BD FACSMelody cell sorter (BD Life Sciences, San Jose, CA) and analyzed with FlowJo software.

### Histology and immunofluorescence analysis

For morphological and histological studies, small sections of each skin substitutes were fixed in formol (Thermo Fisher Scientific, Waltham, MA). Then staining with hematoxylin and eosin was performed on 6 μm thick sections of each skin substitute. For the measurement of the living epidermis thickness, two skin substitutes each from three different populations were measured using ImageJ software (National Institutes of Health, http://imagej.nih.gov/ij). In total, 10 measurements per condition were quantified (N = 3, n = 2, 10 measurements per photo). For immunofluorescence staining, sections of skin substitutes were embedded in OCT compound (Sakura Finetek, Torrance, CA) and frozen in liquid nitrogen until utilization. When needed, 6 μm sections were cut and indirect immunofluorescence staining was performed on each section after 10 min fixation in cold acetone. Primary and secondary antibodies were prepared using PBS containing 1% bovine serum albumin. The slides were incubated for 45 min in a dark chamber at room temperature with the primary antibodies and then for 30 min with the secondary antibodies in the same conditions. The primary and secondary antibodies used are detailed in the supplementary material (see supplemental Table S1). The slides were incubated with a mounting medium containing 4′-6-diamidino-2-phenylindole Fluoromount-G (SouthernBiotech, Birmingham, AL) in order to stain the cell nuclei. Sections of each skin substitute were observed under a Zeiss Axio Imager (Carl Zeiss Canada Ltd., Toronto, ON, Canada).

### Western blot immunoblotting

The dermis and epidermis were separated and then ground into powder using a cryogenic grinder (Cryomill MM400; Retsch®, Newtown, PA). The extraction of protein was performed using a RIPA buffer supplemented with a protease inhibitor cocktail (cOmplete, Roche, Diagnostics GmBH, Germany). Proteins were then separated and resolved by SDS-PAGE on 10% polyacrylamide gels (20 μg of total proteins was used for each sample). Total proteins resolved on the gels were transferred overnight onto PVDF membranes (GE HealthCare, Chicago, IL). For each marking, the membrane was blocked in TBS with the addition of 5% nonfat milk and 0.05% Tween-10 over a period of 1 h. The primary antibodies were incubated with the membrane overnight at 4°C with agitation (excepted for the Fas antibody that was incubated only for 1 h). Then the membranes were incubated with HRP-labeled secondary antibodies for 1 h. The primary and secondary antibodies used are detailed in the supplementary material (see supplemental Table S1). The detection of protein expression was performed using the Amersham ECL Western Blotting Detection Reagent (GE Healthcare, Chicago, IL). A Fusion Fx7 imager (MIB Lab Equipment, Kirkland, QC, CAN) was used to assess protein expression from the membranes, and the quantification of the immunoblots was performed using ImageJ software (National Institutes of Health, http://imagej.nih.gov/ij).

### Enzyme-linked immunosorbent assay

The ELISAs were performed using day 1 air-liquid culture supernatants. The levels of IL-17A and IL-10 were assayed using the IL-17A and IL-10 Competitive ELISA Kits (Thermo Fisher Scientific, Vienna, Austria). A volume of 150 μl of supernatant was used to detect the levels of IL-17A and 100 μl of supernatant was used to detect the levels of IL-10.

### NFκB protein assay

The Proteome Profiler Human NFκB protein array (R&D Systems, Minneapolis, MN) was used following the protocol. A total of 41 human proteins and 4 serine or tyrosine phosphorylation sites were detected by the array. The cell lysates of each condition were obtained after separation of the dermis and epidermis using forceps, and the grinding of the epidermal tissues into powder was done using a cryogenic grinder (Cryomill MM400; Retsch®, Newtown, PA). A total of 400 μg of proteins (a volume of 250 μl) was used to detect the specific proteins on each array membrane. The proteins and serine/tyrosine sites were detected with a Fusion Fx7 over a period of 15 min (MIB Lab Equipment, Kirkland, QC, Canada). The quantification of each array membrane was done using ImageJ software (National Institutes of Health, http://imagej.nih.gov/ij).

### Statistical analysis

All statistical analyses were conducted using R (v.4.3.0) (https://www.R-project.org/) in RStudio (v.2023.06.0) (http://www.posit.co/). Mixed-effects linear models were fit using *lme4* (v.1.1–33) (36). Contrasts were estimated using *emmeans* (v.1.8.6) (37). Model fit evaluations as well as assumption checks were done through visualizations using *performance* (v.0.10.4) (38). Effects were considered significant when the 95% confidence interval (CI) for the estimates did not include 0. Plots were built using Prism (v.9.0 for macOS) (Graphpad, Software, La Jolla, CA).

## Results

### EPA affects the activation levels of T cells in contact with psoriatic keratinocytes

**Fig. 1:**
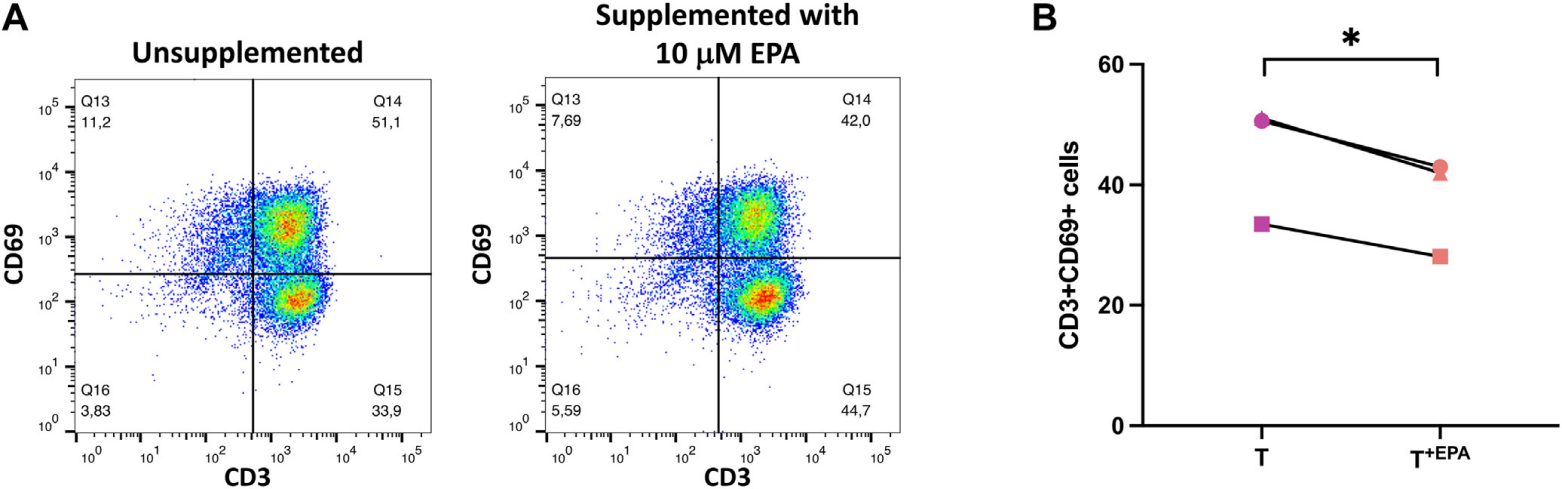
CD69 expression in T cells cocultured with psoriatic keratinocytes in medium supplemented or not with 10 μM EPA A: Flow cytometric analysis of CD3 and CD69 expression in T cells that were cultured with psoriatic keratinocytes in medium supplemented or not with 10 μM EPA (T and T^+EPA^). B: Percentages of CD3- and CD69-positive cells in T cells that were cultured with psoriatic keratinocytes in medium supplemented or not with 10 μM EPA (T and T^+EPA^). N = 3 psoriatic keratinocyte populations. Asterisks indicate CIs that exclude 0. T^+EPA^, T cells cultured in media supplemented with EPA.

In the 2D coculture of psoriatic keratinocytes and T cells, we investigated the expression of CD3 and CD69 by flow cytometry in T cells that were cultured with psoriatic keratinocytes in medium supplemented or not with 10 μM EPA (T and T^+EPA^), in order to evaluate the effects of EPA on T cell activation. The proportion of activated CD3+ and CD69+ T cells (T) in unsupplemented media was approximately 51.1%, while T cells that were cultured in medium supplemented with EPA (T^+EPA^) presented levels of activation of approximately 42%. Therefore, under our culture conditions and accounting for repeated measures, the supplementation of the culture media with EPA decreased the percentage of CD69-positive T cells in coculture with psoriatic keratinocytes by 7.4 points (95% CI [−9.5, −5.3]). At the time of analysis, only about half of the T cells were still positive for the CD69 marker (Fig. 1B).

### Impact of EPA on T cell differentiation and polarization in a coculture with psoriatic keratinocytes

**Fig. 2:**
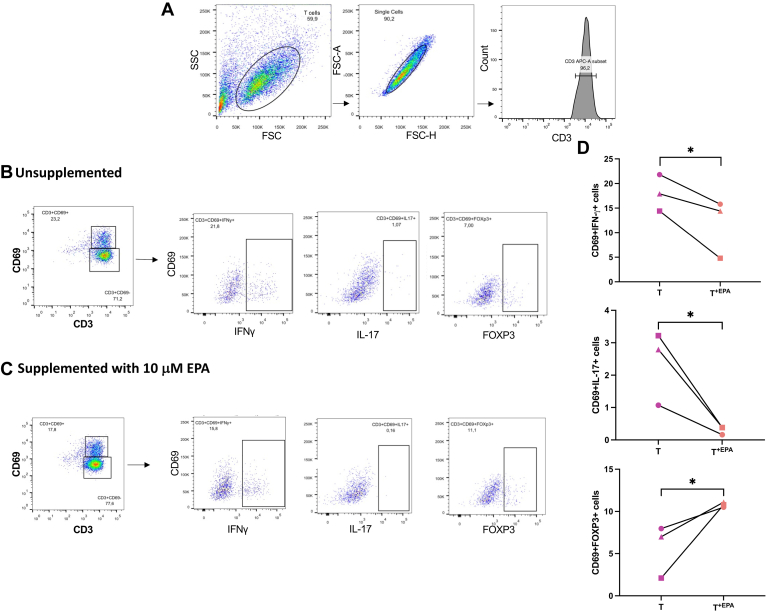
Intracellular marking of IFN-γ, IL-17A, and FOXP3 in T cells cocultured with psoriatic keratinocytes in medium supplemented or not with 10 μM EPA. A: Flow cytometric analysis to identify T cells cocultured with psoriatic keratinocytes in medium supplemented or not with 10 μM EPA according to their size and granularity (forward scatter [FCS] and side scatter [SSC]). B: Flow cytometric analysis of IFN-γ, IL-17A, and FOXP3 expression in T cells cultured in unsupplemented media (T). C: Flow cytometric analysis of IFN-γ, IL-17A, and FOXP3 expression in T cells cultured in media supplemented with 10 μM EPA (T^+EPA^). D: Percentages of intracellular marker (IFN-γ, IL-17A, and FOXP3) and CD69-positive cells in T cells cocultured with psoriatic keratinocytes in medium supplemented or not with 10 μM EPA (T and T^+EPA^). N = 3 psoriatic keratinocyte populations. Asterisks indicate CIs that exclude 0. IL, interleukin; T, T cells; T^+EPA^, T cells cultured in media supplemented with EPA.

In the 2D coculture of psoriatic keratinocytes and T cells, the intracellular marking of inflammatory markers, namely IFN-γ, IL-17A, or the anti-inflammatory marker FOXP3, was performed on T cells that were cultured with psoriatic keratinocytes in medium supplemented or not with 10 μM EPA (T and T^+EPA^), in order to evaluate the influence of EPA on T cell polarization. Under our culture conditions, the supplementation of the culture media with EPA decreased the proportion of T cells positive for CD69 and IFN-γ (CD69+IFN-γ+) as compared with unsupplemented T cells by 6.4 points (95% CI [−9.8, −2.9]) (Fig. 2C). Moreover, the addition of EPA to the coculture of T cells and psoriatic keratinocytes diminished the percentage of CD69+IL17A+ cells in T^+EPA^ compared with T by 2.1 points (95% CI [−3.2, −0.9]) (2.3% positive cells in T as compared with 0.3% positive cells in T^+EPA^) (Fig. 2C). Inversely, the exogenous EPA allowed an increase of 5.1 points (95% CI [1.5, 8.7]) in the proportion of CD69+FOXP3+ cells in T^+EPA^ compared with T (5.7% positive cells in T and 10.8% positive cells in T^+EPA^) (Fig. 2C). Thus, the supplementation of the media with EPA diminished the amounts of IL-17A marking, suggesting a decrease in the proportion of IL-17A–producing T cells, in favor of the FOXP3 marking, which identifies Treg cells. The analyses performed on complete T cells populations (those present in the supernatants and those having adhered to keratinocytes) showed similar results, with a decrease in the proportion of IL-17A–positive cells following EPA supplementation (see supplemental Fig. S4). The labeling of FOXP3 and IL-17A carried out on cocultures of healthy keratinocytes and polarized T cells demonstrated a predominance of the FOXP3 labeling (67% of CD3 and FOXP3-positive cells) over that of IL-17A (0.02% of CD3 and IL-17A–positive cells) (see supplemental Fig. S3).

### Impact of EPA on cutaneous morphology and functionality of the skin substitutes

**Fig. 3:**
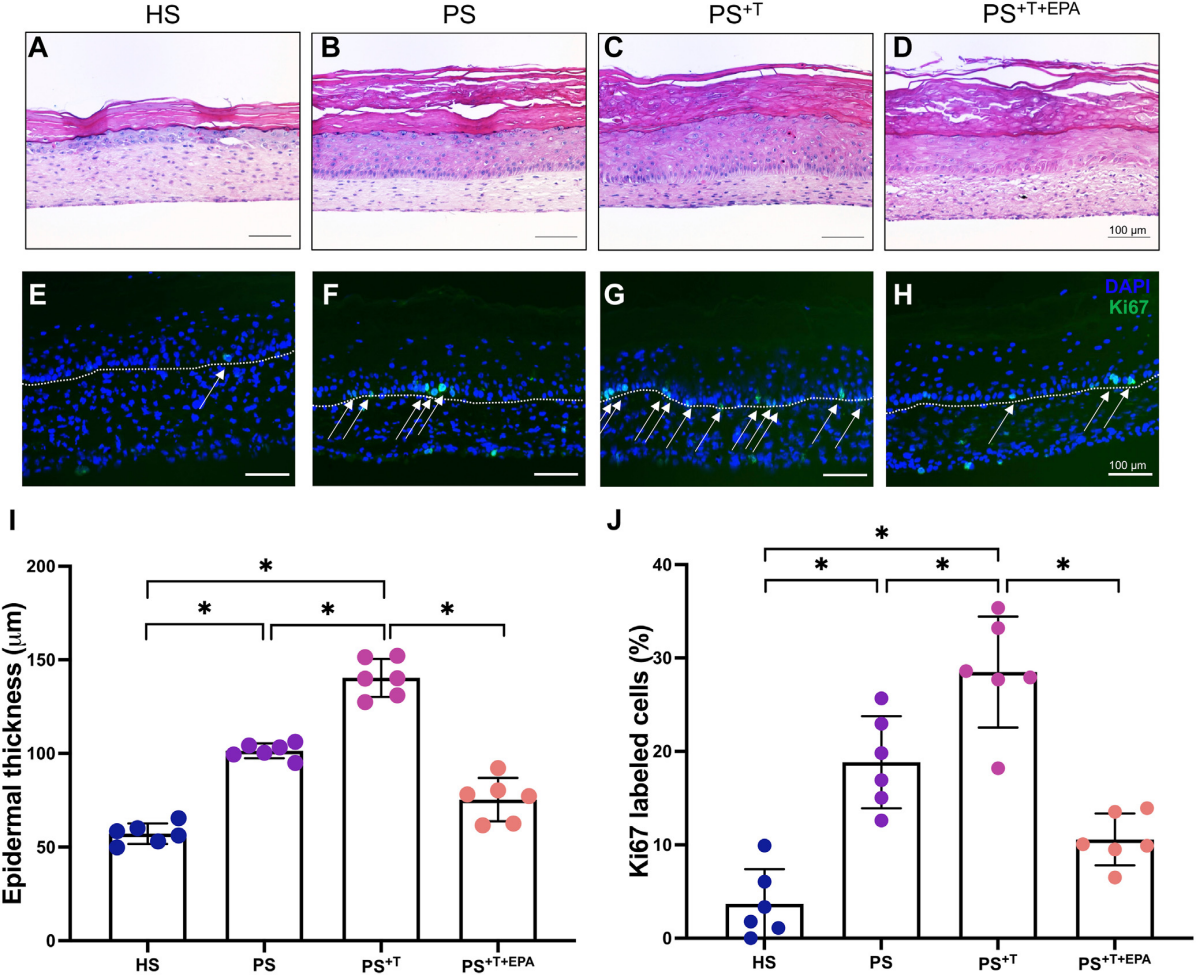
Histological analysis of the skin substitutes. A–D: Histological aspects of hematoxylin and eosin (H&E) staining of healthy and psoriatic skin substitutes. Scale bar represents 100 μm. E–H: Immunofluorescence staining of Ki67 showing basal proliferative keratinocytes in healthy and psoriatic skin substitutes. The arrows show the labeled cells. Scale bar represents 100 μm. DAPI is shown in blue and Ki67 is shown in green. The dotted line shows the dermo-epidermal junction of the skin substitute. I: Thickness of the living epidermis of healthy and psoriatic skin substitutes. J: Percentages of Ki67-positive cells (the number of positive cells over the number of total keratinocytes in the basal layer of each skin substitute). The values are presented as mean ± SD (N = 3 donors, n = 2 skin substitutes per donor). Asterisks indicate CIs that exclude 0. DAPI, 4′-6-diamidino-2-phenylindole; HS, healthy substitutes; PS, psoriatic substitutes; PS^+T^, psoriatic substitutes produced with T cells; PS^+T+EPA^, psoriatic substitutes produced with T cells and supplemented with EPA; T, T cells.

To assess the impact of EPA on the T cell profile in a representative and complete psoriatic model, healthy and psoriatic skin substitutes were produced according to the self-assembly model using culture media supplemented or not with 10 μM EPA. The histological analyses of the skin substitutes showed that psoriatic substitutes (PS) and PS produced with T cells (PS^+T^) presented an increased living epidermis thickness by 44.2 μm (95% CI [−58.2, −30.2]) and 83.1 μm (95% CI [−97.1, −69.2]), respectively compared with the healthy skin substitute control (HS), demonstrating the acanthosis found in native psoriatic skin (Fig. 3A–C and I). The addition of polarized T cells to the psoriatic model also increased the epidermal thickness of the substitutes, suggesting that T cells play a role in the proliferation of lesional keratinocytes (Fig. 3B, C and I). The epidermis of PS produced with T cells and supplemented with EPA (PS^+T+EPA^) was not as thick as PS^+T^, indicating a decrease in acanthosis in the presence of EPA, even in the psoriatic model produced with polarized T cells (Fig. 3C, D and I). PS^+EPA^ also presented a smaller living epidermis (see supplemental Fig. S5). The expression of Ki67, which is a proliferation marker that identifies proliferative basal keratinocytes, was evaluated in the different skin substitute conditions (39). PS and PS^+T^ had more basal keratinocytes positive for Ki67 compared with HS, which is representative of the hyperproliferation found in psoriatic skin (Fig. 3E–G, I and J). Nonetheless, an increase was observed in the number of Ki67-positive cells in PS^+T^ compared with PS, suggesting that polarized T cells participate in the activation of keratinocyte proliferation (Fig. 3F, G and J). The supplementation of the culture media with EPA in PS^+T+EPA^ helped normalize the abnormal cell proliferation of psoriatic keratinocytes, as observed in the decreased expression of Ki67 found in basal keratinocytes (Fig. 3H and J). Ki67 was also decreased in the PS^+EPA^ condition (see supplemental Fig. S5). In addition, EPA generally promoted a modulation in some hallmark psoriatic markers, mainly psoriasin and elafin (see supplemental Fig. S6).

### EPA slows down the incorporation of T cells and modulates the secretion of an inflammatory cytokine in the psoriatic skin model

**Fig. 4:**
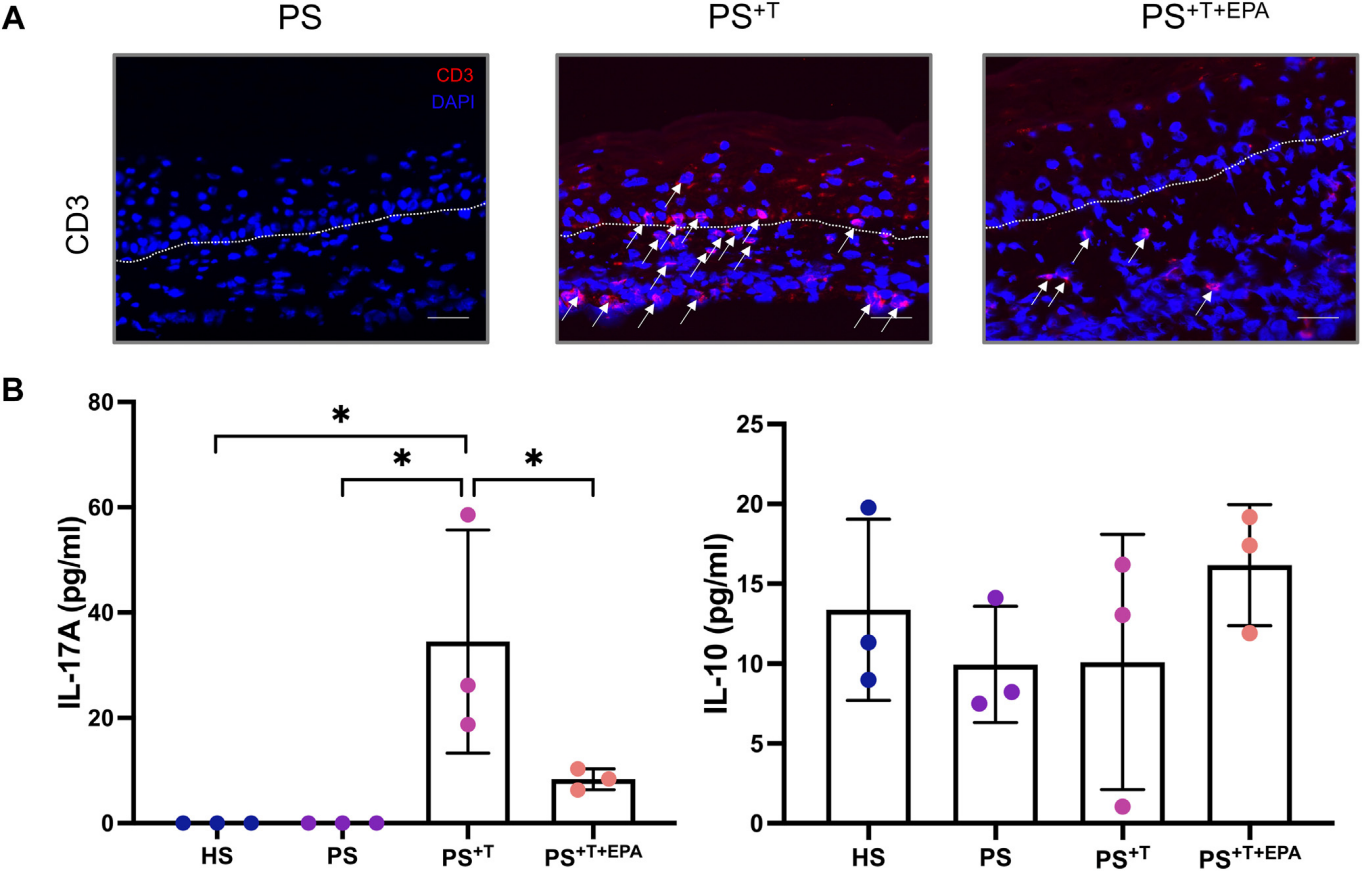
Expression of a T cell marker and secretion of key psoriatic cytokines in psoriatic skin substitutes. A: Indirect immunofluorescence staining was performed on PS, PS^+T^, and PS^+T+EPA^. CD3 expression is represented in red. The cell nuclei were counterstained with DAPI reagent and are represented in blue. The arrows show the CD3-labeled cells and the dashed white lines represent the basement membrane. 20× magnification. Scale bars: 100 μm. B: IL-17A and IL-10 levels in the culture supernatants of the skin substitutes (N = 3 donors, n = 1 culture supernatant per donor). Asterisks indicate CIs that exclude 0. DAPI,4′-6-diamidino-2-phenylindole; HS, healthy substitutes; IL-10, interleukin-10; IL-17A, interleukin-17A; PS, psoriatic substitutes; PS^+T^, psoriatic substitutes produced with T cells; PS^+T+EPA^, psoriatic substitutes produced with T cells and supplemented with EPA.

To correctly confirm the incorporation of T cells in our psoriatic skin model, an indirect immunofluorescence staining was performed to identify the expression of the specific T cell marker CD3 (Fig. 4A). The presence of T cells in PS^+T^ and PS^+T+EPA^, observable with the CD3 labeling, illustrated well the ability of polarized T cells to integrate and migrate in the tissues of the psoriatic skin model. However, the CD3-labeling found in PS^+T^ seemed stronger than in the PS^+T+EPA^ condition, suggesting a retention of T cells adhering to PS following EPA supplementation (Fig. 4A). Moreover, the levels of cytokines involved in psoriasis and, respectively, characteristic of IL-17A–producing T cells and Treg cells (IL-17A and IL-10) were measured in the culture supernatants of HS, PS, PS^+T^, and PS^+T+EPA^ using independent ELISA assays to characterize the T cell profile of each condition (Fig. 4B). The amount of IL-17A was higher in PS^+T^ as compared with HS and PS by 34.5 pg/ml for both conditions (95% [−54.5, −14.5] and [−56.5, −12.5], respectively), which is representative of a proinflammatory environment. The addition of exogenous EPA to PS^+T+EPA^ diminished the overproduction of IL-17A in PS produced with polarized T cells (Fig. 4B). As for IL-10, the secretion of this anti-inflammatory cytokine was at its highest in PS^+T+EPA^ when compared with the other psoriatic conditions. However, we did not detect an effect on IL-10 levels when adding T cells to PS^+T^ and PS^+T+EPA^. In addition, EPA had no impact on IL-10 levels in PS (PS^+EPA^) as well as in PS^+T+EPA^ (Fig. 4B and see supplemental Fig. S5).

### The supplementation of the culture media with EPA decreases the phosphorylation of STAT proteins in the dermis

**Fig. 5:**
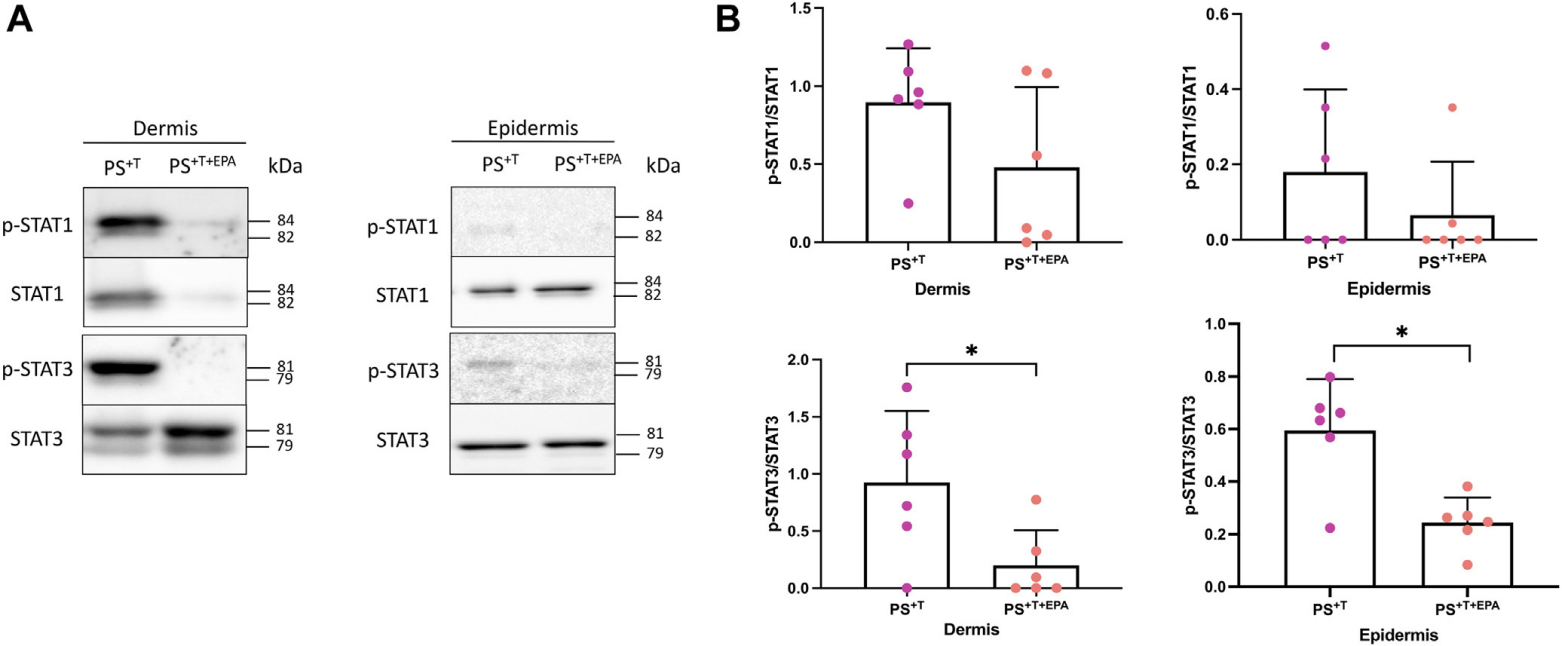
Expression of STAT proteins in psoriatic skin substitutes. A: Twenty micrograms of total protein from skin substitutes was analyzed by immunoblot for the presence of STAT1 and STAT3 proteins as well as their phosphorylated forms (p-STAT1 and p-STAT3) in the epidermis and the dermis of PS^+T^ and PS^+T+EPA^. β actin was used to control equal loading. One representative immunoblot is shown per protein. (N = 3 donors per condition; n = 2 skin substitutes per donor). B: Semiquantitative analysis of panel A. Asterisks indicate CIs that exclude 0. PS^+T^, psoriatic substitutes produced with T cells; PS^+T+EPA^, psoriatic substitutes produced with T cells and supplemented with EPA; STAT, signal transducer and activators of transcription; T, T cells.

To support the results obtained in the coculture 2D model regarding T cell polarization, Western blot–type immunoblots were performed to semiquantify the levels of phosphorylation of STAT1 and STAT3 proteins in the dermis and epidermis of PS^+T^ and PS^+T+EPA^, since STAT1 allows Th1 cell differentiation, while Th17 cell development requires signaling through STAT3 (14). Phosphorylated protein expression was not detectable in skin substitutes without T cells (HS and PS, data not shown). In the dermis, the phosphorylated form of STAT1 and STAT3 (p-STAT1 and p-STAT3) were mostly detected in PS^+T^ as compared with PS^+T+EPA^. In fact, the p-STAT3/STAT3 ratio was decreased by 0.7 (95% CI [−1.3, −0.1]) following the supplementation of the culture media with EPA in the dermis of PS^+T+EPA^ (Fig. 5B). EPA had no effect on p-STAT1 expression in the dermis (Fig. 5B). As for the epidermis, the expression of the phosphorylated form of these proteins was practically undetectable, showing a weak migration of T cells in the epidermis of the skin substitutes, especially in the skin substitutes produced with exogenous EPA (Fig. 5A and B). Despite the low proportion of these proteins in the epidermal fractions of the skin substitutes, a decrease in the level of STAT3 phosphorylation was measured in PS^+T+EPA^ epidermis as compared with PS^+T^ with a ratio of p-STAT3/STAT3 decreased by 0.4 (95% CI [−0.5, −0.2]) (Fig. 5B).

### Impact of EPA supplementation on the expression levels of the NFκB signaling pathway proteins in the psoriatic skin model

**Fig. 6:**
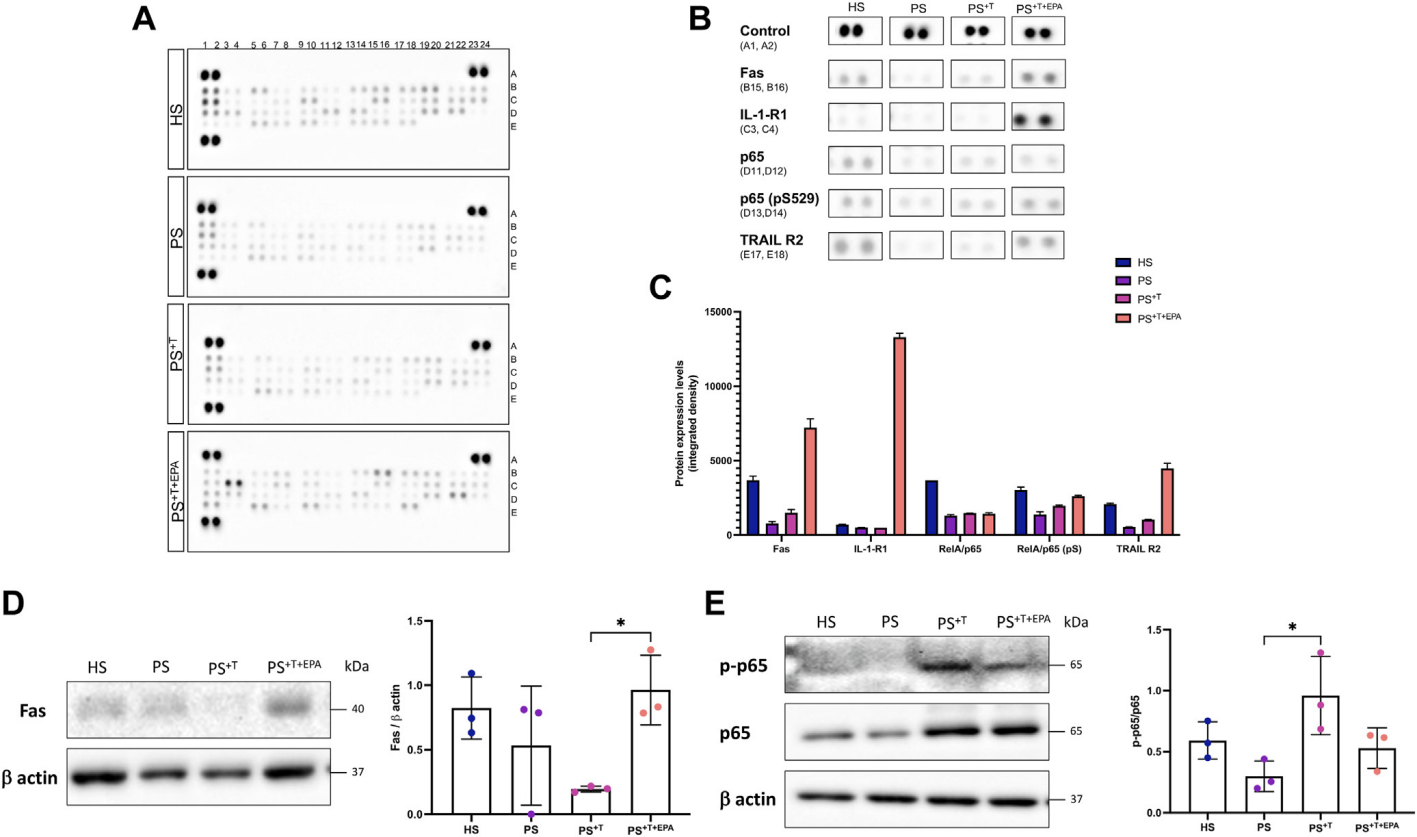
Levels of proteins related to the NFκB pathway in HS, PS, PS^+T^ and PS^+T+EPA^. A: Cell lysates from HS, PS, PS^+T^, and PS^+T+EPA^ (400 μg) were used to detect the expression of NFκB-related proteins using the Proteome Profiler Human NFκB Pathway Array kit. B: Close-up spots from panel (A) corresponding to the proteins whose expression was the most affected by the EPA supplementation. C: Densitometric analysis of the dot blot duplicates from panel (A) (N = 1 cell population, n = 1 cell lysate per population). D: Twenty micrograms of total proteins from skin substitutes was analyzed by immunoblot for the presence of Fas in the epidermis of HS, PS, PS^+T^, and PS^+T+EPA^. β actin was used to control equal loading. One representative immunoblot is shown per protein (N = 3 donors per condition; n = 1 skin substitute per donor). E: Twenty micrograms of total proteins from skin substitutes was analyzed by immunoblot for the presence of p65 and its phosphorylated form in the epidermis of HS, PS, PS^+T^, and PS^+T+EPA^. β actin was used to control equal loading. One representative immunoblot is shown per protein (N = 3 donors per condition; n = 1 skin substitute per donor). Asterisks indicate CIs that exclude 0. HS, healthy substitutes; PS, psoriatic substitutes; PS^+T^, psoriatic substitutes produced with T cells; PS^+T+EPA^, psoriatic substitutes produced with T cells and supplemented with EPA; T, T cells.

In order to detail more precisely the mechanism of action by which EPA modulates the T cell response in the psoriatic model, the expression levels of proteins involved in the NFκB signaling pathway were measured using the Proteome Profiler Human NFκB pathway array kit (Fig. 6A, B). The vast majority of proteins were detected in HS, PS, PS^+T^, and PS^+T+EPA^. The levels of expression of the proteins related to the pathway were mostly reduced in PS and PS^+T^ as compared with HS. In counterpart, the supplementation with EPA in PS^+T+EPA^ slightly enhanced the expression of most of the proteins involved in the NFκB pathway, thus approaching the expression levels found in HS, which is observable in the close-up of Fig. 6B. Important changes following EPA supplementation in PS^+T+EPA^ were found in the proteins involved in the regulation of cell death (ASC, Fas, TNFRI, TRAIL R2), which mostly belong to the non-canonical NFκB pathway. The detection of the proteins Fas and TRAIL R2 was increased in PS^+T+EPA^ compared with other skin conditions, suggesting the implication of EPA in triggering T cell apoptosis (Fig. 6A–C). These results were confirmed by Western blot analysis where an increase in the expression of Fas was observed in PS supplemented with EPA (Fig. 6D, see supplemental Fig. S5). TRAIL R2 expression was undetectable by Western blot (data not shown). Additionally, a slight modulation of p65 expression was noted following EPA supplementation (Fig. 6B, C). Immunoblot analysis showed an increase of p65 phosphorylation in PS^+T^ as compared with PS, while EPA tended to downregulate the expression of p-p65 in PS^+T+EPA^ (Fig. 6E). EPA did not impact p65 expression in PS (see supplemental Fig. S5). However, the most substantial alteration measured after EPA supplementation was observed in IL-1-R1 expression, with an extensive upregulation observed in PS^+T+EPA^ (Fig. 6A–C).

## Discussion

Psoriasis is an autoimmune skin disease that has been widely studied over the years. T cells are at the center of the pathogenesis since they initiate and maintain the development of psoriatic plaques and a high prevalence of Th17 cells is found in psoriatic skin (2, 7). Over the years, many studies have evaluated the impact of n-3 PUFAs on T cell polarization in diverse inflammatory diseases using dietary supplements (27, 40, 41, 42). However, the effects of such long-chain PUFAs on T cell polarization in the human psoriatic disease are still little studied in the scientific field. Our findings showed that the supplementation of the culture media with EPA in a coculture of psoriatic keratinocytes and polarized T cells decreased the number of IL-17A–producing T cells, while increasing the proportion of cells expressing FOXP3. In addition, EPA normalized the proliferation of psoriatic keratinocytes in the 3D psoriatic tissue–engineered model and downregulated the expression of specific psoriatic markers. These effects could be mediated through the activation of proteins related to the non-canonical NFκB signaling pathway, especially Fas and TRAIL R2, which mediate T cell apoptosis, as well as through the modulation of the p65 subunit, which is an essential factor for the canonical pathway of the cascade.

In the present study, flow cytometric analyses demonstrated that approximately 50% of activated and polarized T cells cultured in the presence of psoriatic keratinocytes were still active after a culture period of over 9 days, confirming the effectiveness of the proinflammatory environment generated by psoriatic keratinocytes in maintaining T cells in an active state. Shin *et al.* (43) also demonstrated that the psoriatic epidermis is involved in the promotion of CD69 upregulation. T cells cultured in the presence of healthy keratinocytes appeared to be predominantly Treg cells since they were mainly marked by FOXP3. Inversely, the coculture of psoriatic keratinocytes and polarized T cells (T monolayer) in unsupplemented media showed a prevalence of IL-17A and IFN-γ–positive T cells, representing inflammatory conditions. These results are in line with psoriatic skin constructs, showing a strong Th1-Th17 cell profile after 2 and 5 days of cell culture (43, 44). However, the percentage of CD69+IFN-γ+ and CD69+IL-17A+ cells remained particularly weak despite the polarization of T cells (18.04% and 2.36% positive cells, respectively). These low yields could be explained by the strong cell-cell contact between psoriatic keratinocytes and T cells, as well as their adhesion to the monolayer of keratinocytes. In the past, our team has shown that in an *in vitro* culture of psoriatic keratinocytes and T cells, the chemokines and cytokines secreted by psoriatic keratinocytes strongly promote the adhesion of T cells to keratinocytes (45). However, the flow cytometry analyses were also performed on complete populations of T cells (those present in the supernatants and those having adhered to the keratinocytes), but the cell stripping method greatly affected the expression of surface T cell markers, making the results difficult to interpret since the CD3 labeling allowing the identification of T cells was not clear in the flow cytometry results. It is now recognized that trypsin and other cell detachment methods can lead to changes in cell membrane structure and composition, thus affecting the expression of T cell surface markers (46). It was also found that psoriatic keratinocytes express adhesion molecules and that the addition of cytokines to the culture medium improves the adhesion of T cells to keratinocytes (47, 48). In the present study, in order to not falsify the cytometric analyses with diverse cell types (T cells and keratinocytes), the cytometric labeling was carried out only on the T cells harvested in the supernatants of the 2D culture. Regarding the effects of EPA, it did not reduce the degree of T cell activation, suggesting that in contact with psoriatic keratinocytes, EPA cannot counteract the activity of PMA and ionomycin. The studies by Jaudszus *et al.* and Zeyda *et al.* showed that the expression of CD69 was unaltered in activated T cells after EPA and DHA supplementations (49, 50). This could be explained by the method used for activating T cells. n-3 PUFAs appear to suppress T cell proliferation if activated with CD3/CD28 beads, but not if activated through the use of PMA (51). Herein, EPA restored the profile of T cells to the disadvantage of IL-17A–producing T cells with a reduction in IL-17A marking, confirming that EPA affects the secretory capacity of these cells. This decrease in the proportion of IL-17A–positive cells following EPA supplementation indicates a diminution in the number of IL-17A–producing T cells in the presence of EPA. These results are consistent with the increased proportion of FOXP3 expression in T^+EPA^, proposing a switch in the Th cell balance toward a characteristic profile of mainly Treg cells after EPA supplementation. Monk *et al.* were among the first to explore the effects of an n-3 PUFA–rich diet on T cell polarization in mice, as they have shown that EPA and DHA reduce Th17 polarization in healthy models as well as in disease models such as colitis (27, 41, 52). Since then, many teams have investigated the power of n-3 PUFAs on T cell functions, even though this remains little known in psoriasis. In fact, high EPA levels were associated with the inhibition of Th17 cell differentiation in rheumatoid arthritis *in vitro* and with a restoration of Th17 and Treg balance in collagen-induced arthritis (53, 54). More recently, a randomized study by Kolobaric *et al.* established that the dietary supplementation with n-3 PUFAs through hen eggs in healthy subjects shifts the T cells toward an anti-inflammatory profile, with decreased numbers of peripheral Th17 cells (55). Herein, the upregulation of Treg cells in the EPA-supplemented coculture indicates that there may be an increased immune tolerance in the presence of EPA and therefore a possible return to skin homeostasis. One of the greatest limitations of our study is related to the power of the study which might be affected by the small number of samples and which may prevent us from detecting certain differences between our conditions. However, since the production of PS is technically very challenging and requires more than two months of cell culture, the use of 3 different cell populations is sufficient to perform statistical analyses. It must however be kept in mind that more statistical differences could have been observed with a larger sample size (56).

Since cells cultured in a 3D model may behave differentially than in a monolayer culture that has a very high proliferative capacity, the effects of EPA on the profile of T cells added to the 3D psoriatic model were subsequently evaluated (57). Herein, psoriatic substitutes produced with polarized T cells (PS^+T^) showed a marked thickening of the living epidermis as compared with other conditions, evidence of increased keratinocyte proliferation, which was also confirmed by a high ratio of Ki67-positive cells in PS^+T^. The addition of polarized T cells amplified the acanthosis in the disease, as seen elsewhere (58). The T cells added to the model in PS^+T^ migrated into the epidermis and adopted a secretory profile representative of IL-17A–producing T cells with a strong secretion of IL-17A, as in the unsupplemented coculture model (T monolayer), highlighting the prevalence of these immune cells in the disease. These inflammatory features instigated by polarized T cells in the 3D psoriatic model were also illustrated in our team’s most recent paper (34). It is now proven beyond any doubt that the secretion of IL-17A is essential in the pathogenesis since it triggers keratinocyte proliferation, stimulates angiogenesis, and recruits mast cells and neutrophils to the inflamed skin (59, 60). Inversely, IL-10 was little detected in PS and PS^+T^, which agrees with the data found in the literature. IL-10 reduces the immune response and normalizes keratinocyte maturation in the skin (61, 62, 63). Moreover, the expression of p-STAT1 and p-STAT3 was elevated in the dermis of PS^+T^, analogous with the results of the T cell secretory profile and contributing to the activation loop of psoriasis. Overexpression of the active form of STAT3 in psoriasis leads to increased numbers of IL-17–producing cells and mediates the production of IL-23, resulting in the amplification of the Th17 pathway, while STAT1 stimulates the upregulation of keratin 17 expression (14, 64).

Further findings of this study were the reduction of psoriatic characteristics following EPA supplementation in the 3D psoriatic model. For instance, the addition of EPA normalized the psoriatic phenotype with the reduction of keratinocyte hyperproliferation and a diminution in the production of the proinflammatory cytokine IL-17A, confirming the antipsoriatic potential of EPA even in the 3D culture. The impact of n-3 PUFAs on cytokine production has been widely studied in different inflammatory models, but to our knowledge, our team was the first to investigate it in a human *in vitro* model of psoriasis (31, 65, 66, 67). Quin *et al.* (68) demonstrated that endogenous n-3 PUFAs decreased IL-17 levels in a mouse model of psoriasis. Supplements of 2 g per day of EPA and DHA in healthy subjects were shown to decrease the production of TNF-α, IL-1, and IL-6 by healthy mononuclear cells (69, 70). N-3 PUFA bioactive lipid derivatives also modulate cytokine secretion since resolvin E3 neutralized IL-17 and IL-23 levels in allergic airway inflammation and resolvin D5 lowered IL-1β in a type 1 diabete animal model (71, 72). In addition to studying the effects of EPA on the secretory power of T cells added to the psoriatic model, we showed that EPA impacts the expression of proteins that intrinsically influence the polarization of T cells, namely by decreasing the dermal phosphorylation of STAT3. By doing so, EPA blocked the proliferation of IL-17A–producing T cells, allowing Treg cells to take over and multiply in order to rapidly reduce inflammation (73). The effects of n-3 PUFAs on the expression of transcription factors implicated in T cell signaling are still little investigated. However, Wang *et al.* demonstrated that in prostate cancer cells, the phosphorylation of STAT1 was suppressed by an EPA/DHA treatment (74). Interestingly, EPA seemed to decrease the total STAT1 expression in the dermis of PS^+T+EPA^, whereas normally the non-phosphorylated form of this protein is constitutively expressed. This downregulation could possibly be caused by the capacity of n-3 PUFAs to activate the suppressor of cytokine signaling proteins, which are negative regulators of janus kinase/STAT signaling (75).

The NFκB signaling pathway plays a role in the regulation of T cells and their differentiation as it can contribute to the inactivation of autoreactive T cells (76). Our results mainly showed a decrease in the amounts of proteins associated to this signaling pathway in psoriatic conditions compared with those in healthy conditions, pointing to a diminution of this cascade in a psoriatic environment. No consensus is clearly stated in the literature regarding the expression of the NFκB pathway in psoriasis. While some studies have established that the pathway is overexpressed in the psoriatic context, others have indicated that it is rather decreased in expression (77, 78, 79). In this study, the addition of EPA to the medium elevated the levels of most of the NFκB-related proteins, bringing them closer to the levels found in healthy substitutes, suggesting a diminution of the inflammation. Specifically, elevated amounts of Fas, IL-1R-1, and TRAIL R2 were detected in PS^+T+EPA^ compared with PS and PS^+T^, implying a more pronounced activation of the non-canonical pathway (76). The increase in IL-1R-1 in PS^+T+EPA^ was not expected since studies have shown that this receptor was strongly activated in psoriasis-like skin inflammation in an imiquimod-induced mouse model (80). However, the expression detected by the array represents the basal levels of IL-1R-1 and does not illustrate the activated form of the receptor. Moreover, Bebes *et al.* (81) determined that IL-1R-1 expression was higher in Treg cells, especially in psoriatic skin. A diminution of the non-canonical pathway is associated with a reduced formation of medullary thymic epithelial cells, allowing the generation of autoimmune reactions (82). In addition, non-canonical NFκB plays a pro-apoptotic role with Fas being an essential activator that leads to clonal deletion of effector T cells (76). To assess the implication of EPA on the canonical pathway, we measured the levels of p65 and its phosphorylated form p-p65 in the different conditions. An increase in p-p65 was detected in PS^+T^, which could correlate with upregulation transactivation of the target genes of diverse activities, including cellular proliferation and production of inflammatory cytokines and chemokines (83). EPA tended to modulate the phosphorylation of p65 in PS^+T+EPA^, suggesting a downregulation of the canonical pathway. Some data indicated that following the activation of the canonical receptor, EPA could retain p50 and p65 subunits in the cytoplasm, thus inhibiting the whole downstream cascade (84). Some previous studies also suggested that n-3 PUFAs could decrease NFκB activation in monocytes and macrophages, respectively, but studies remain unclear regarding the involvement of essential fatty acids in the complete NFκB pathway (85, 86). Therefore, EPA seems to support cutaneous immune homeostasis and prevents the recruitment and uncontrolled activation of leukocytes through the NFκB non-canonical pathway, while potentially reducing the inflammation engendered by the canonical pathway via reduced p65 phosphorylation (87).

## Conclusion

Altogether, our results indicate that EPA can favorably attenuate the features of psoriasis that are modulated by T cells. Flow cytometric analyses of T cells cocultured with psoriatic keratinocytes showed that EPA affects T cell polarization with a decrease in the number of IL-17A–producing T cells accompanied by an increase in FOXP3-positive T cells, symbolizing a better immune tolerance following EPA supplementation. The added EPA advantageously modulated the overproliferation of psoriatic keratinocytes in the *in vitro* psoriatic model produced with polarized T cells, emphasizing the capacity of EPA to diminish the inflammatory environment even in a more complex model. Our study also illustrated the capacity of EPA to alter the inflammatory functions of T cells that were incorporated in the psoriatic model, mainly by decreasing their migration into the epidermis as well as their secretory power with a strong downregulation of IL-17A and the diminution of dermal STAT3 phosphorylation. Finally, we showed that EPA modifies the expression of proteins related to the NFκB signaling pathway, and that this modulation could explain the high prevalence of Treg cells in PS supplemented with EPA. While our results add to the data on the effects of n-3 PUFA on the NFκB signaling pathway in psoriasis, further research will be needed to shed light on this matter since current studies remain incomplete. In addition, a more in-depth study of the mechanisms of action of n-3 PUFAs in psoriasis, including the involvement of their lipid mediators such as 18-hydroxyeicosapentaenoic acid will allow further assessment of their total antipsoriatic potential.

## Data availability

All data are contained within the manuscript. Raw data and scripts used for analysis are available upon request.

## Supplemental data

This article contains supplemental data (88, 89).

## Conflict of interest

The authors declare that they have no conflicts of interest with the contents of this article.
