# Supplementary material for: Eicosapentaenoic acid reduces the proportion of IL-17A–producing T cells in a 3D psoriatic skin model
Source: J Lipid Res. 2023 Aug 18;64(9):100428. doi: 10.1016/j.jlr.2023.100428 (PMC10509711; doi:10.1016/j.jlr.2023.100428)
Supplement: Supplemental Figures S1–S6 and Table S1 [file mmc1.docx]

**Supplementary material**


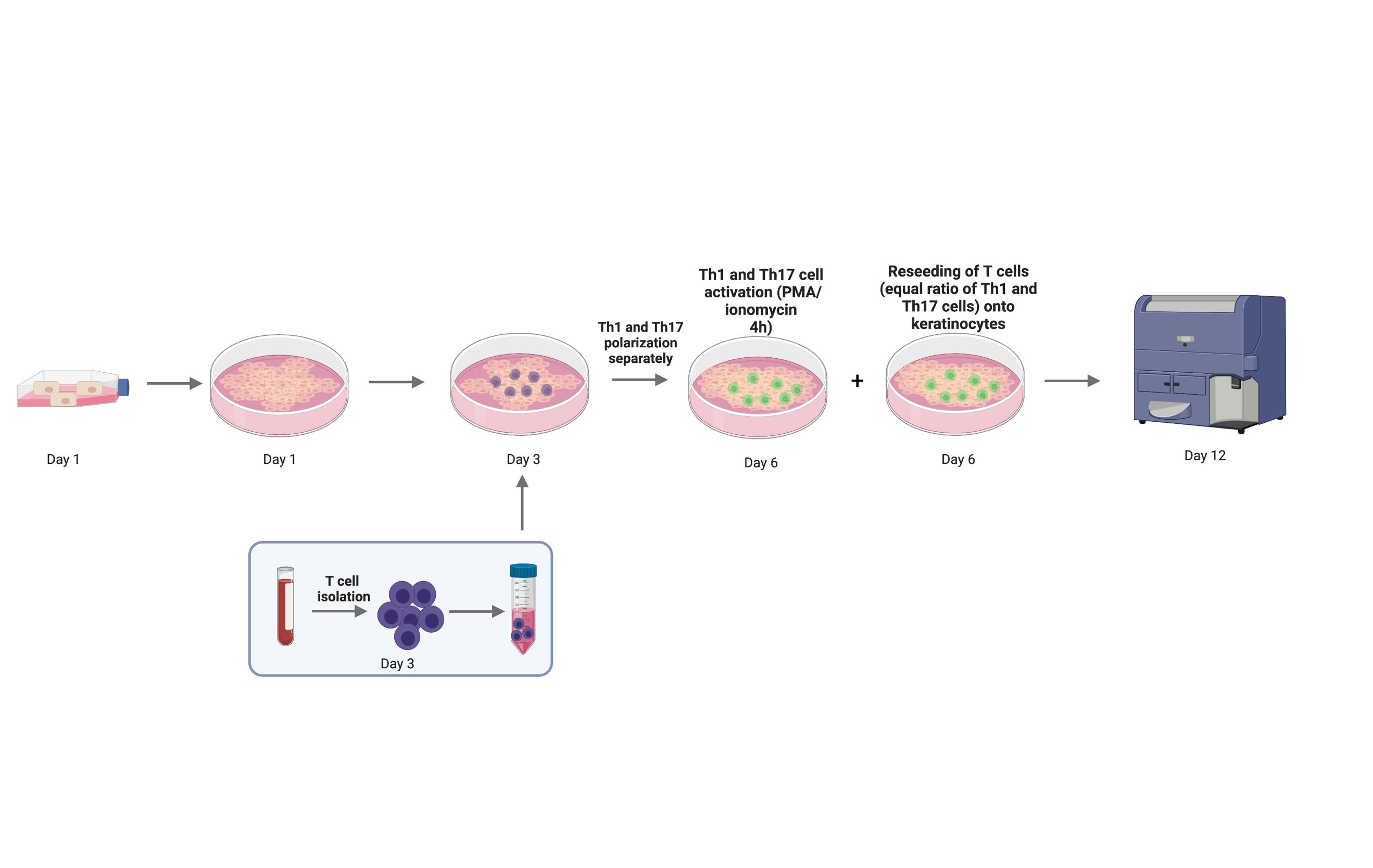


**Figure S1. Co-culture of psoriatic keratinocytes and T cells.** Keratinocytes were seeded onto a layer of irradiated human fibroblasts and cultivated in medium supplemented or not with 10 μM EPA. At day 3, T cells were isolated and seeded onto the keratinocytes and separately polarized towards either the Th1 or the Th17 phenotype. Then the Th1 and Th17 cells were separately activated and re-seeded onto keratinocytes at an equal ratio (50/50) of Th1 and Th17 cells and cultivated for 6 more days in medium supplemented or not with 10 μM EPA. The T cells were then analyzed by flow cytometry.

CD4 and CD8 marking was performed on T cells after their isolation with the EasySep method, in order to clearly distinguish the proportion of each cell type after isolation. The proportion of cells positive for CD4 was approximately 65%, while that for CD8 was about 30%.


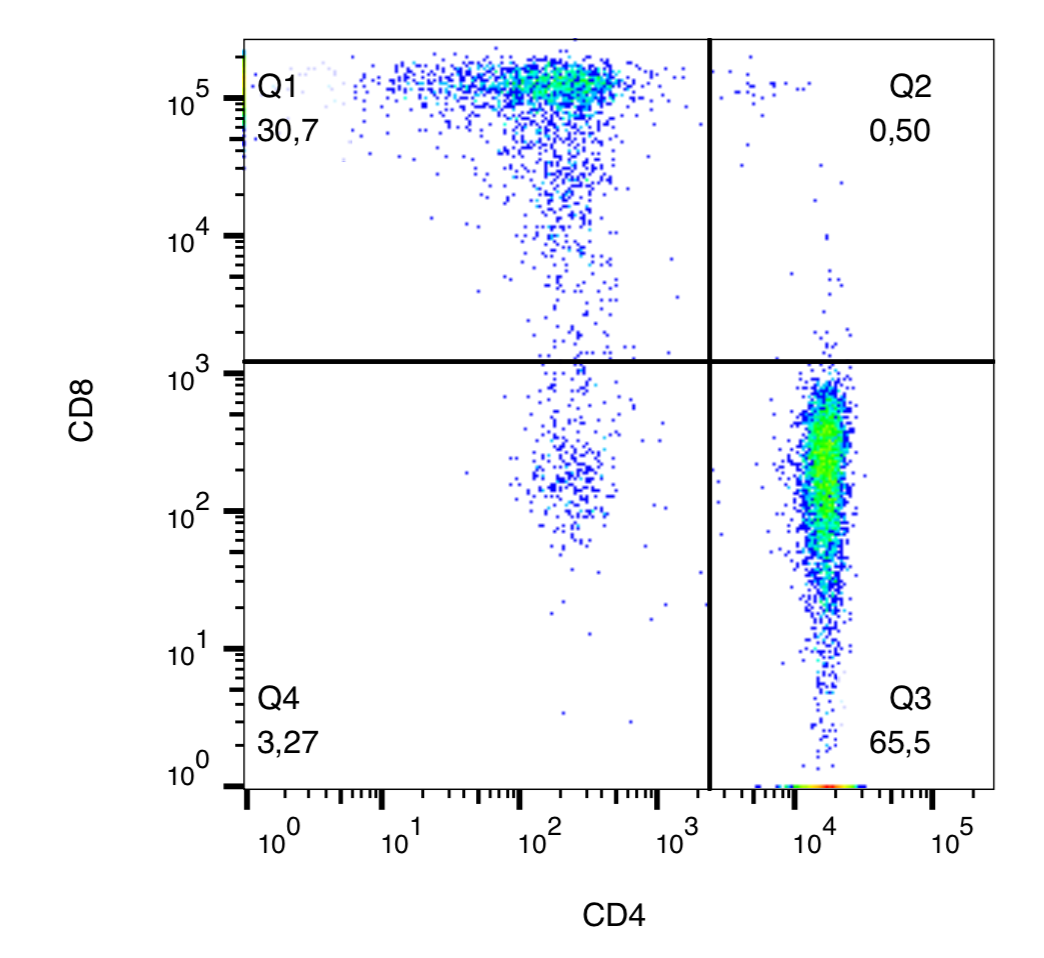


**Figure S2. CD4 and CD8 labeling after T cell isolation.** Flow cytometric analysis of T cells after isolation with the EasySep method. Analysis of CD4 and CD8 expression.

CD69 marking as well as intracellular marking of markers specific to different sub-populations of Th cells (Th17 and Treg cells), namely IL-17A and FOXP3, were performed on T cells that were cultivated in co-culture with healthy keratinocytes in medium supplemented or not with 10 μM of EPA (T and T^+EPA^), in order to evaluate the influence of healthy keratinocytes on T cell activation and polarization. The proportion of cells positive for CD69 was practically undetectable (0.2% only), suggesting that healthy keratinocytes do not provide and environment conducive to maintaining T cells in an active state. The number of T cells positive for FOXP3 and IL-17A was measured in regard of cells positive for CD3 since the activation levels of T cells was too low. The proportion of T cells positive for CD3 and FOXP3 (CD3+FOXP3+) was elevated (67.0% of positive cells) while that of IL-17A (CD3+IL-17A+) was very weak (0.02% of positive cells). Thus, T cells that were cultivated in co-culture with healthy keratinocytes adopt a profile characteristic of Treg cells.


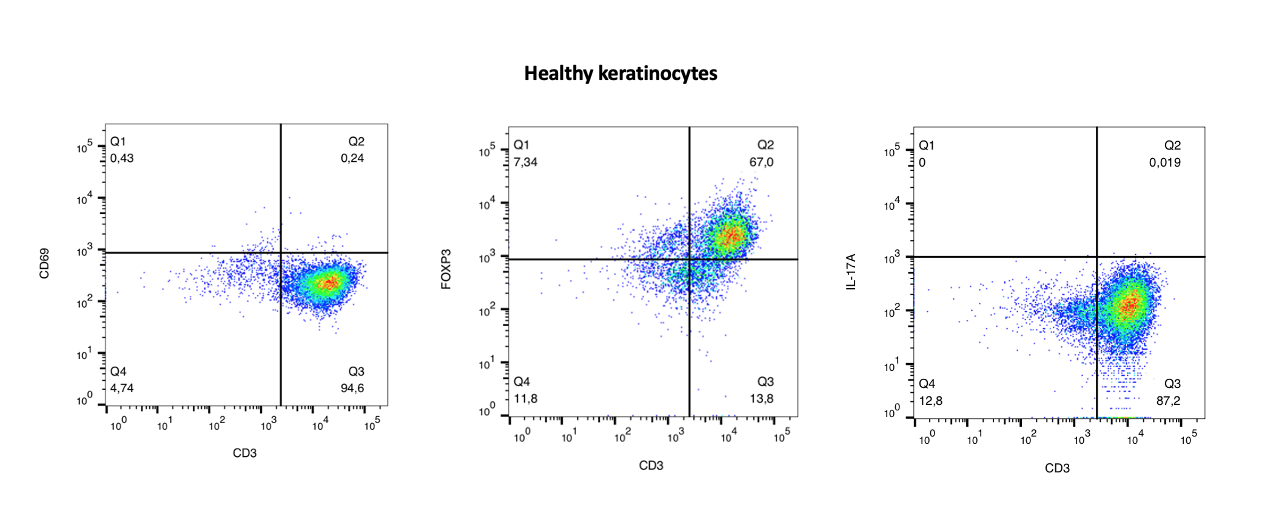


**Figure S3. Intracellular marking of CD69, FOXP3 and IL-17A in monolayer of healthy keratinocytes cultured with T cells in unsupplemented medium.** Flow cytometric analysis of T cells after stimulation with PMA/ionomycin cultivated in unsupplemented medium. Analysis of CD3 with CD69, FOX-P3 and IL-17A expression separately**.**

The intracellular staining of markers specific to different sub-populations of T cells (Th1, Th17 and Treg), namely IFN-γ, IL-17A and FOXP3, was performed on complete populations of T cells (those present in the supernatants and those having adhered to keratinocytes) that were cultivated with psoriatic keratinocytes in medium supplemented or not with 10 μM EPA (T and T^+EPA^). The detachment method used to remove the T cells affected the surface markers, namely CD3 and CD69, thus reducing the number of T cells available for the analysis. Distinguishing the different populations that are positive for the labels is difficult. However, the intracellular labeling was performed on the small cell population recovered and the results showed that the addition of EPA reduced the proportion of CD69+IL-17A+ cells in T^+EPA^ compared with T (9.8% positive cells in T compared with 3.6% positive cells in T^+EPA^) (Figure S4). Similarly, the EPA supplementation lowered the proportion of IFN-γ positive cells in T^+EPA^ (11.6% positive cells in T compared with 6.1% positive cells in T^+EPA^) (Figure S4). We did not detect any effect of the addition of EPA on the proportion of FOXP3-positive complete T cells (Figure S4).

**
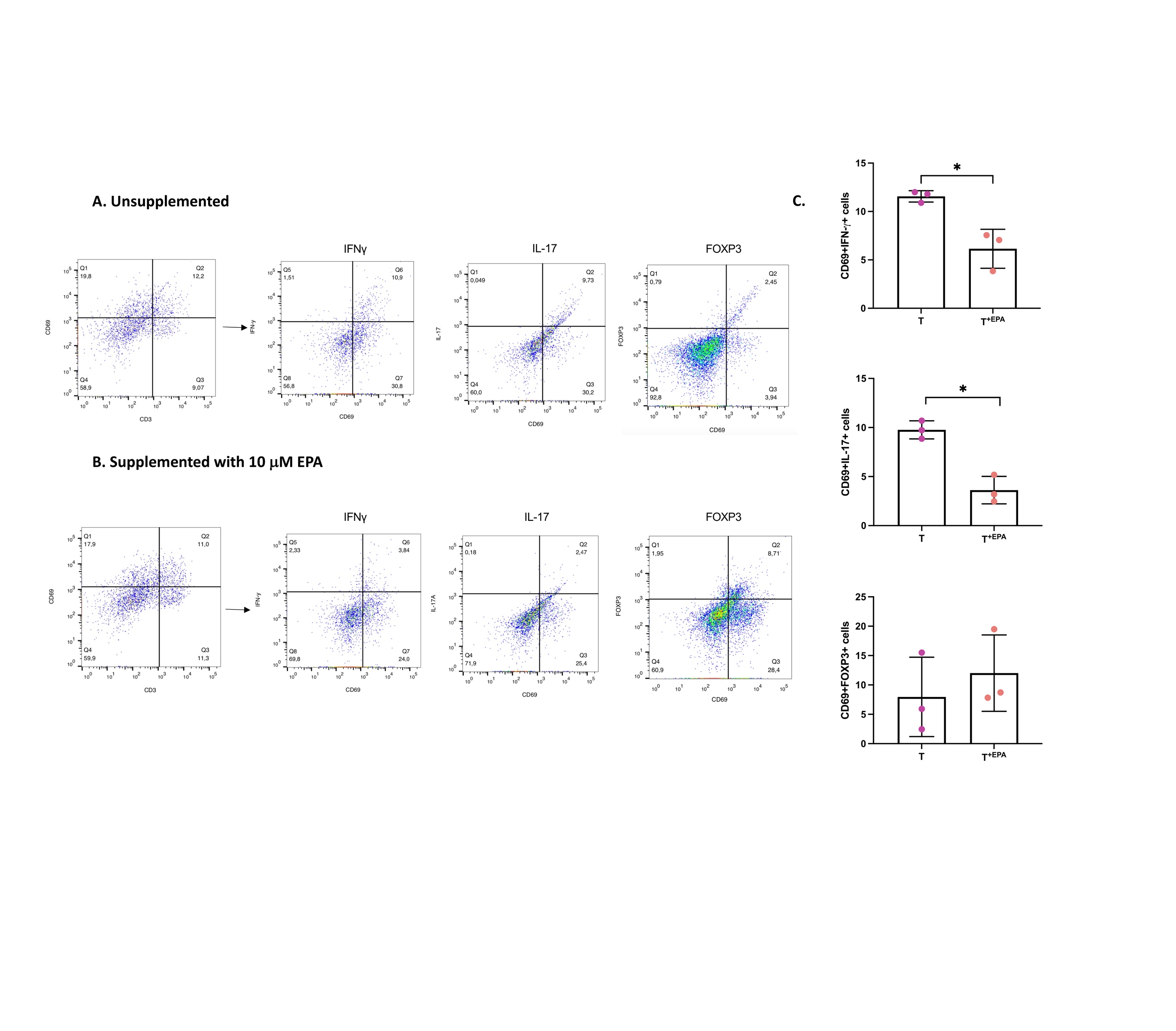
**

**Figure S4. Intracellular marking of IFN-γ, IL-17A and FOXP3 in complete populations of T cells co-cultured with psoriatic keratinocytes in medium supplemented or not with 10 μM EPA. (A)** Flow cytometric analysis of IFN-γ, IL-17A and FOXP3 expression in complete populations of T cells cultivated in unsupplemented media (T); **(B)** Flow cytometric analysis of IFN-γ, IL-17A and FOXP3 expression in complete populations of T cells cultivated in media supplemented with 10μM EPA (T^+EPA^); **(C)** Percentages of intracellular marker (IFN-γ, IL-17A and FOXP3) and CD69 positive cells in complete populations of T cells co-cultured with psoriatic keratinocytes in medium supplemented or not with 10 μM EPA (T and T^+EPA^). N=3 psoriatic keratinocyte populations. Asterisks indicate CIs that exclude 0.

The histological analyses of the skin substitutes HS, PS, PS^+T^ and PS^+T+EPA^ are described in the manuscript. The epidermis of PS^+EPA^ was not as thick as PS, indicating a decrease in acanthosis in the presence of EPA (Figure S4). The expression of Ki67 in PS^+EPA^ was decreased compared with PS, more representative of the Ki67 levels found in HS (Figure S5). The levels of cytokines involved in psoriasis and respectively characteristic of Th17 and Treg cells (IL-17A and IL-10) were measured in the culture supernatants of HS, PS, PS^+EPA^, PS^+T^ and PS^+T+EPA^ using independent ELISA assays to characterize the T cell profile of each condition. The levels found in HS, PS, PS^+T^ and PS^+T+EPA^ are presented in the manuscript. IL-17A was not detected in the culture media alone nor in PS^+EPA^ since no T cells were added to this condition (data not shown). As for IL-10, the addition of EPA to PS^+EPA^ did not affect the secretion of this anti-inflammatory cytokine in psoriatic substitutes (Figure S5). IL-10 was not detected in the culture media alone (data not shown). The expression levels of proteins involved in the NFκB signaling pathway were measured by western-blot analysis in HS, PS, PS^+EPA^, PS^+T^ and PS^+T+EPA^. The levels quantified in HS, PS, PS^+T^ and PS^+T+EPA^ are presented in the manuscript. The EPA supplementation had no effect on the expression of Fas in PS^+EPA^. As for the levels of phosphorylation of p65, no changes were detected in the psoriatic substitutes supplemented with EPA (PS^+EPA^) (Figure S5).


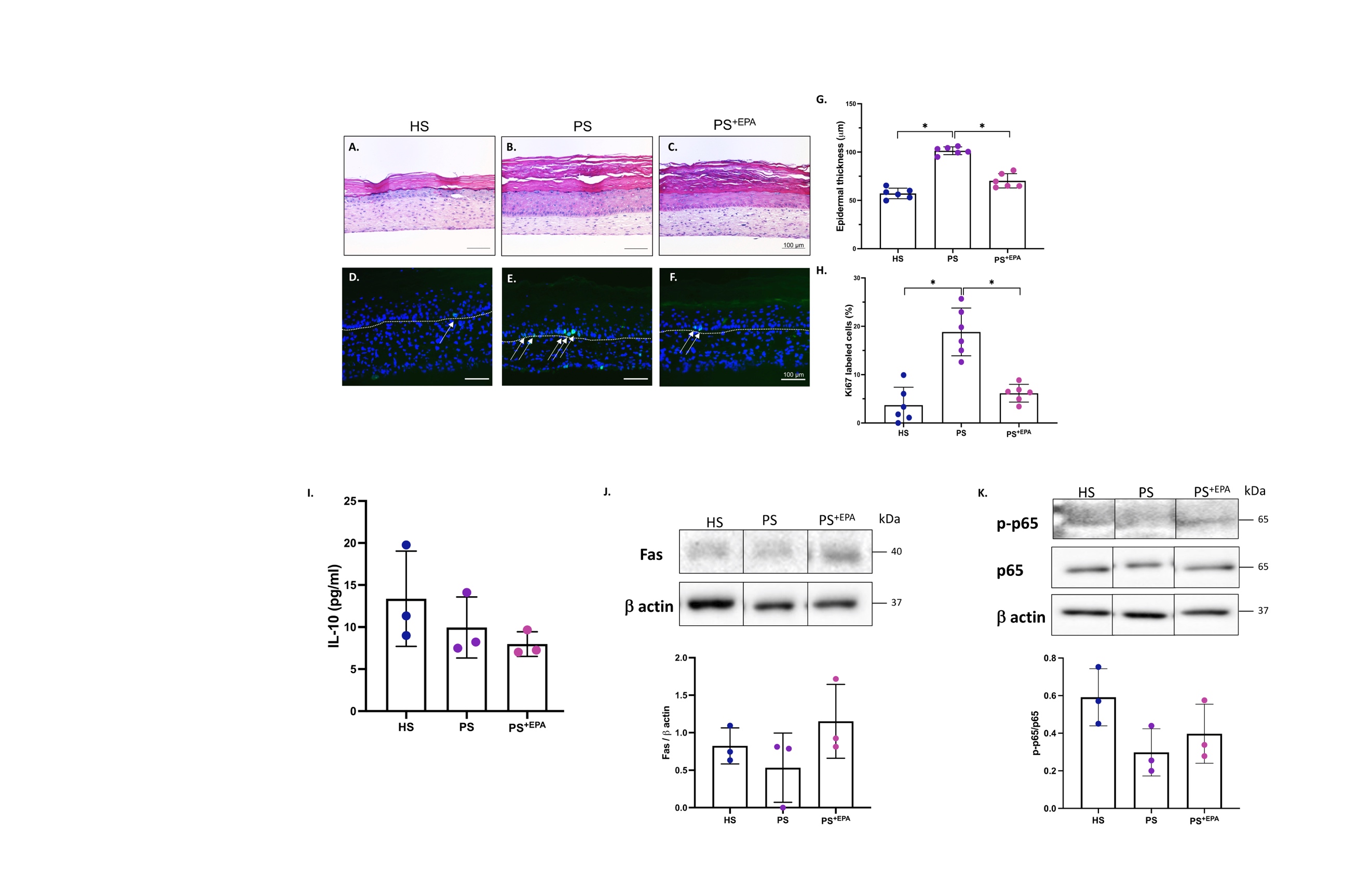


**Figure S5. Histological analysis, secretion of cytokines and levels of proteins related to the NFκB pathway of the skin substitutes. (A-C)** Histological aspects of hematoxylin and eosin (H&E) staining of the skin substitutes (HS, PS and PS^+EPA^). A and B are reuse from Figure 3A and B in order to clearly highlight the effects of EPA herein in C. Scale bar represents 100 μm; **(D-F)** Immunofluorescence staining of Ki67 showing basal proliferative keratinocytes in the skin substitutes (HS, PS and PS^+EPA^). D and E are reuse from Figure 3E and F in order to clearly highlight the effects of EPA herein in F. The arrows show the labeled cells. Scale bar represents 100 μm. DAPI is shown in blue and Ki67 is shown in green. The dotted line shows the dermo-epidermal junction of the skin substitute; **(G)** Thickness of the living epidermis of the skin substitutes (HS, PS and PS^+EPA^); **(H)** Percentages of Ki67 positive cells (the number of positive cells over the number of total keratinocytes in the basal layer of each skin substitute); **(I)** IL-10 levels in the culture supernatants of the skin substitutes (HS, PS, PS^+EPA^); **(J)** Twenty micrograms of total protein from skin substitutes was analyzed by immunoblot for the presence of Fas, in the epidermis of HS, PS, PS^+EPA^. HS and PS conditions were reuse from Figure 6D in order to clearly highlight the effects of EPA herein. β actin was used to control equal loading. Densitometric analyses represent the data of all 3 donors; **(K)** Twenty micrograms of total protein from skin substitutes was analyzed by immunoblot for the presence of p65 and its phosphorylated form p-p65 in the epidermis of HS, PS, PS^+EPA^. HS and PS conditions were reuse from Figure 6E in order to clearly highlight the effects of EPA herein. β actin was used to control equal loading. Densitometric analyses represent the data of all 3 donors. The values are presented as mean ± SD (N=3 donors, n=2 skin substitutes per donor (western-blot analyses=1 skin substitute per donor)). Asterisks indicate CIs that exclude 0. Abbreviations: HS: healthy substitutes; EPA: eicosapentaenoic acid; PS: psoriatic substitutes; PS^+EPA^: psoriatic substitutes supplemented with EPA; PS^+T^: psoriatic substitutes produced with T cells; PS^+T+EPA^: psoriatic substitutes produced with T cells and supplemented with EPA; T: T cells.

Immunofluorescence staining were performed to analyze and detect the expression of psoriatic-specific markers in the different skin substitute conditions. Elafin is an inhibitor of leucocyte elastase and proteinase 3 and its expression is upregulated in psoriatic epidermis, contributing to the infiltration of neutrophils into the skin (1). In our psoriatic model (PS), the expression of elafin seemed to be increase when compared with the healthy control (HS). Additionally, PS^+T^ presented a more uniform and accentuated distribution of the protein in the substitute as compared with the control. As for EPA supplementation, the levels of elafin were disturbed in PS^+EPA^ and PS^+T+EPA^ (Figure S6). Psoriasin (S100A7) is involved in cell progression and proliferation of psoriatic keratinocytes, as seen in PS and PS^+T^ with elevated expression (2). However, the localization of the expression of this protein in the skin substitutes supplemented with EPA (PS^+EPA^ and PS^+T+EPA^) did not allow to draw any conclusion regarding the effects of EPA (Figure S6).

**
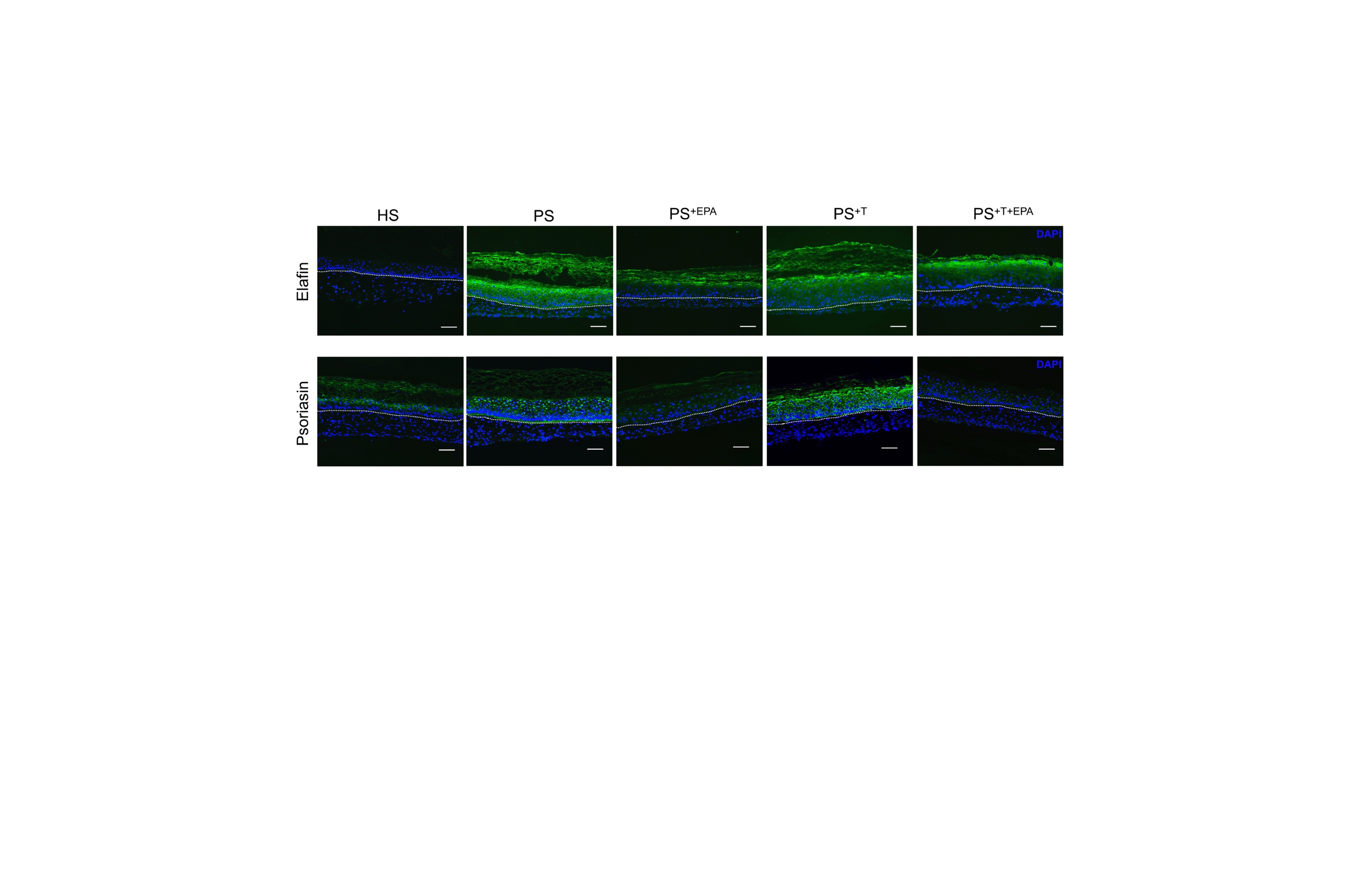
**

**Figure S6. Expression of psoriatic markers.** Indirect immunofluorescence staining was conducted on HS, PS, PS^+EPA^, PS^+T^ and PS^+T+EPA^. Elafin and psoriasin expression are represented in green. The cell nuclei were counterstained with DAPI reagent and are represented in blue. The dashed white lines represent the basement membrane. Scale bars: 100 µm.

Table S1. Complete list of antibodies used for indirect immunofluorescence and western blot analyses

| **Antigens** | **Source** | **Company** | **Catalog number** | **Lot number** | **Dilution** |
| --- | --- | --- | --- | --- | --- |
| ***Primary antibodies*** | | | | | |
| **FITC anti-human CD69** | Mouse monoclonal IgG1 | Biolegend | 310904 | B319620 | 1/20 |
| **APC anti-human CD3** | Mouse monoclonal IgG1 | Biolegend | 300312 | B273399 | 1/20 |
| **PE anti-human IL-17A** | Mouse monoclonal IgG1 | Biolegend | 512306 | B255234 | 1/20 |
| **PE-Cy7 anti-human IFN-γ** | Mouse monoclonal IgG1 | Biolegend | 506517 | B293213 | 1/20 |
| **PE anti-human FOXP3** | Rat monoclonal IgG2a | Invitrogen | 12-4776-42 | 2189793 | 1/20 |
| **Anti-human Ki67** | Mouse monoclonal IgG1 | BD Biosciences | 556003 | 8072956 | 1/400 |
| **Anti-human elafin** | Rabbit polyclonal | Abcam | Ab46774 | GR3224968-1 | 1/100 |
| **Anti-human psoriasin** | Rabbit | Abcam | Ab83534 | GR14369-1 | 1/300 |
| **Anti-human CD3** | Mouse monoclonal IgG1 | Biolegend | 344820 | B240446 | 1/100 |
| **Anti-human STAT1** | Rabbit monoclonal | Cell signaling technology | 14994T | 4 | 1/1000 |
| **Anti-human STAT3** | Rabbit monoclonal | Cell signaling technology | 126405 | 4 | 1/1000 |
| **Anti-human p-STAT1** | Rabbit monoclonal | Cell signaling technology | 7649 | 5 | 1/500 |
| **Anti-human p-STAT3** | Rabbit monoclonal | Cell signaling technology | 9131S | 30 | 1/500 |
| **Anti-human Fas** | Rabbit monoclonal | Cell signaling technology | 4233 | 5 | 1/500 |
| **Anti-human p65** | Rabbit polyclonal | Abcam | Ab16502 | GR3440283-1 | 1/6000 |
| **Anti-human phospho-p65** | Rabbit polyclonal | Invitrogen | 44-711G | 2492272 | 1/500 |
| **Anti-actin beta** | Mouse monoclonal | Sigma | A5441 | 079M4799V | 1/30 000 (epidermis) and 1/10 000 (dermis) |
| ***Secondary antibodies*** | | | | | |
| **Anti-rabbit Alexa Fluor 488** | Donkey polyclonal IgG | Life Technologies | A21206 | 1754421 | 1/1600 |
| **Anti-mouse Alexa Fluor 488** | Goat polyclonal IgG | Life Technologies | A11001 | 1890503 | 1/1600 |
| **Streptavidin Alexa Fluor 594** | Conjugate | Life Technologies | S-11227 | 65B2-1 | 1/1000 |
| **Anti-rabbit HRP** | Goat polyclonal IgG | Jackson ImmunoResearch Laboratories | 111-035-003 | - | 1/60 000 |
| **Anti-mouse HRP** | Goat polyclonal IgG | Jackson ImmunoResearch Laboratories | 115-005-003 | - | 1/60 000 |

**References**

1. Nonomura K, Yamanishi K, Yasuno H, Nara K, Hirose S. Up-regulation of elafin/SKALP gene expression in psoriatic epidermis. J Invest Dermatol. 1994;103(1):88-91.

2. Ekman AK, Vegfors J, Eding CB, Enerback C. Overexpression of Psoriasin (S100A7) Contributes to Dysregulated Differentiation in Psoriasis. Acta Derm Venereol. 2017;97(4):441-8.
